# The Association between Motivation, Affect, and Self-regulated Learning When Solving Problems

**DOI:** 10.3389/fpsyg.2017.01346

**Published:** 2017-08-08

**Authors:** Martine Baars, Lisette Wijnia, Fred Paas

**Affiliations:** ^1^Department of Psychology, Education and Child Studies, Erasmus University Rotterdam Rotterdam, Netherlands; ^2^Roosevelt Center for Excellence in Education, HZ University of Applied Sciences Middelburg, Netherlands; ^3^Early Start Research Institute, University of Wollongong, Wollongong NSW, Australia

**Keywords:** affect, motivation, mental effort, self-regulated learning, problem-solving performance

## Abstract

Self-regulated learning (SRL) skills are essential for learning during school years, particularly in complex problem-solving domains, such as biology and math. Although a lot of studies have focused on the cognitive resources that are needed for learning to solve problems in a self-regulated way, affective and motivational resources have received much less research attention. The current study investigated the relation between affect (i.e., Positive Affect and Negative Affect Scale), motivation (i.e., autonomous and controlled motivation), mental effort, SRL skills, and problem-solving performance when learning to solve biology problems in a self-regulated online learning environment. In the learning phase, secondary education students studied video-modeling examples of how to solve hereditary problems, solved hereditary problems which they chose themselves from a set of problems with different complexity levels (i.e., five levels). In the posttest, students solved hereditary problems, self-assessed their performance, and chose a next problem from the set of problems but did not solve these problems. The results from this study showed that negative affect, inaccurate self-assessments during the posttest, and higher perceptions of mental effort during the posttest were negatively associated with problem-solving performance after learning in a self-regulated way.

## Introduction

Problem-solving is an important cognitive process, be it in everyday life, at work or at school. Problem-solving is the process in which people put effort into closing the gap between an initial or current state (also called givens) and the goal state (Mayer, 1992; Jonassen, 2011; Schunk, 2014). Research has shown that self-regulated learning (SRL) skills are important for effective problem-solving (e.g., Ackerman and Thompson, 2015). Self-regulated learning can be defined as “the degree to which learners are metacognitively, motivationally, and behaviorally active participants in their own learning process” (Zimmerman, 2008, p. 167). Not surprisingly, SRL skills like monitoring and regulating learning processes are important for learning during school years and in working life (Winne and Hadwin, 1998; Zimmerman, 2008; Bjork et al., 2013; for a meta-analysis see, Dent and Koenka, 2016). The process by which learners use SRL skills such as monitoring and control in reasoning tasks, problem-solving, and decision-making processes is also called meta-reasoning (Ackerman and Thompson, 2015). Monitoring judgments about problem-solving tasks and decision-making processes could be related to the effort learners put into finding and using different types of strategies to solve the problem or make a decision.

Self-regulated learning skills are especially important in learner-controlled, online learning environments in which students need to be able to accurately keep track of their own learning process (i.e., monitoring) and have to make complex decisions about what problem-solving task to choose next during their learning process (i.e., regulation choices). Apart from the high cognitive demands imposed by SRL, which have been investigated frequently in previous research (e.g., Dunlosky and Thiede, 2004; Griffin et al., 2008; Van Gog et al., 2011), learning to solve problems in a self-regulated way also imposes demands on affective and motivational resources (Winne and Hadwin, 1998; Pekrun et al., 2002; Spering et al., 2005; Efklides, 2011; Mega et al., 2014). The current study investigated the role of affect and motivation in learning problem-solving tasks in a complex learner-controlled online learning environment for secondary education students.

### Learning to Solve Problems

There are many different kinds of problem-solving tasks, varying from well-structured transformation problems that have a clearly defined goal and solution procedure, to ill-structured problems that do not have a well-defined goal or solution procedure (Jonassen, 2011). In educational settings like schools, universities, or trainings, students usually solve well-structured problems, especially in the domains of science, technology, engineering, and mathematics (STEM domains). Although, well-structured problems, such as math and biology problems encountered in primary and secondary education, can typically be solved by applying a limited and known set of concepts, rules, and principles, they are considered complex in terms of the high number of interacting elements that needs to be considered simultaneously in working memory (WM) during the problem-solving process (e.g., Kalyuga and Singh, 2016).

For learning to solve such complex problems, it is efficient to “borrow” and “reorganize” knowledge of others (Sweller and Sweller, 2006) by learning from examples, such as worked examples and modeling examples (Van Gog and Rummel, 2010). A worked example is a step-by-step worked-out solution to a problem-solving task that students can study. Research in the context of cognitive load theory (CLT; Paas et al., 2003a; Sweller et al., 2011) has shown that for novices, studying worked examples of how the problem should be solved, is a more effective strategy for learning to solve problems than solving equivalent conventional problems (i.e., the worked example effect; Sweller and Cooper, 1985; Paas, 1992; for reviews see, Sweller et al., 1998). According to CLT, having learners study worked examples is an effective way to reduce the extraneous load that is imposed by conventional problem-solving, because the learner can devote all available WM capacity to studying the worked-out solution and constructing a schema for solving such problems in long-term memory (Paas and Van Gog, 2006). In a modeling example, an adult or peer model performing a task can be observed, either face to face, on video, via a screen recording made by the modeling person, or as an animation (Van Gog and Rummel, 2010). According to social-cognitive theory (Bandura, 1986), the learner can construct a mental representation of the task that is being modeled, and use it to perform the task at a later point in time.

According to the resource-allocation framework by Kanfer and Ackerman (1989) and CLT (Sweller et al., 1998) it can be assumed that the competition for WM resources between learning to solve a problem and self-regulation processes can have negative effects on either or both of these processes. For example, a student working on a complex problem-solving task needs most cognitive resources to perform the task itself, which leaves little resources to monitor and regulate learning. During the learning process, it could therefore be beneficial to study worked examples. Studying the step-by-step explanation on how to solve the problem leaves more WM resources for the construction of cognitive schemas (i.e., learning) than solving problems (i.e., worked example effect; Sweller, 1988; for reviews see, Sweller et al., 1998; Van Gog and Rummel, 2010). Therefore, it can be expected that the surplus cognitive capacity that becomes available by the reduction of extraneous cognitive load can be devoted to activities that further contribute to learning performance, such as self-regulation processes (Paas and Van Gog, 2006).

Despite this expectation, SRL skills, such as monitoring one’s own learning processes, have been found to be suboptimal when studying worked examples (Baars et al., 2014a,b, 2016). A possible reason for this finding is that students’ monitoring process when learning from worked examples can be prone to an illusion of competence. Students overestimate their competence to solve a problem when information about the problem solution is present during studying (Koriat and Bjork, 2005, 2006; Bjork et al., 2013). Similarly, studies with primary and secondary education students have found that students who learned to solve problems by studying worked examples showed inaccurate monitoring performance, because they overestimated their future test performance (Baars et al., 2013, 2014a,b, 2016; García et al., 2015). Yet, accurate monitoring is a prerequisite for effective self-regulation (cf. Thiede et al., 2003), and plays an important role in learning to solve problems (Mayer, 1992; Zimmerman and Campillo, 2003).

In a previous study by Kostons et al. (2012) video models were used to explain to secondary education students how to solve hereditary problems and additionally used the video-modeling examples to train students to self-assess their performance and make regulation choices in a learner-controlled environment. In the study, problem-solving performance, self-assessment, and task selection accuracy improved. These results are promising. However, large standard deviations in self-assessment accuracy and task selection were found, suggesting large individual differences in these SRL skills (Kostons et al., 2012), indicating that some students benefitted more from the video-modeling examples than others. Among others, Kostons et al. (2012) have suggested that these differences might be explained by motivation and affect.

### Problem-Solving, Affect, and Motivation

Students’ affect and motivation can facilitate or hinder students when learning to solve problems in a self-regulated way. Affect was found to influence the use of different strategies (e.g., organization of study time, summarizing materials), SRL activities (e.g., reflecting on learning), and motivation; all factors that can impact academic achievement (Pekrun et al., 2002; Efklides, 2011; Mega et al., 2014). Moreover, in the domain of problem-solving, positive and negative affect were found to influence the problem-solving strategies (e.g., seeking and use of information) that students used (Spering et al., 2005).

According to theories on SRL both affect and motivation play an important role in SRL (e.g., Winne and Hadwin, 1998; Pintrich, 2004; Efklides, 2011). According to Efklides (2011), the interaction between metacognition, motivation, and affect is the basis of students’ SRL. In Efklides’ Metacognitive and Affective model of SRL (MASRL model), SRL is not only determined by a person’s goal, but also by an interaction between metacognitive experiences, motivation, and affect during task performance. In line with the MASRL model, a study by Mega et al. (2014) showed that both negative and positive affect influence different aspects of SRL. For instance, positive affect was positively related to the evaluation of learning performance and metacognitive reflection during studying. In addition, both negative and positive affect were also shown to influence students’ motivation. For example, positive affect enhanced students’ beliefs on incremental theory of intelligence and their academic self-efficacy. Positive affect was found to have a greater impact on both SRL abilities and motivation compared to negative affect. SRL abilities and motivation in turn were predictive of academic achievement. However, the effect of motivation on academic achievement was larger than the effect of SRL abilities on academic achievement. Mega et al. (2014) further showed that the relation between affect and academic achievement was mediated by motivation and SRL abilities.

Although the study by Mega et al. (2014) showed the influence of affect on motivation and SRL abilities and subsequent academic performance, the implications for learning a variety of subjects during school years are still not clear. In the study by Mega et al. (2014), two general academic achievement indicators were used with undergraduate students from different disciplines. These general indicators of academic achievement were productivity (i.e., number of exams passed) and ability (i.e., GPA). These indicators are domain general and therefore, it is unclear whether these results would also apply to the domain of problem-solving or to task-specific performance within a domain.

#### The Role of Affect in Problem-Solving

In the domains of problem-solving and decision-making, it was found that positive affect facilitates flexible and creative thinking, and decision-making in complex environments such as medical decision-making (Fiedler, 2001; Isen, 2001). In a review by Isen (2001), it was shown that if the situation is important or interesting to a person, positive affect will enhance systematic, cognitive processing and thereby make this process more efficient and innovative. Positive affect was found to improve generosity, creativity, variety seeking, negotiation, and decision-making in a range of different domain and contexts such as problem-solving (e.g., Duncker’s problem), consumer decision-making, coping with stressful life-events, bargaining when buying and selling appliances, car choice, and medical diagnosis. For example, physicians with positive affect induced by a small gift (i.e., a box of candy), scored higher on creativity as measured by the Remote Associates Test (Estrada et al., 1994). Also, in a study by Politis and Houtz (2015) it was found that middle school students who watched a positive video program to induce positive affect generated a greater number of ideas compared to students who watched a neutral video program. More closely related to problem-solving tasks that can be solved in a stepwise manner, Brand et al. (2007) demonstrated that affect influenced solving the Tower of Hanoi (ToH) problem in adult students. After inducing negative affect, participants needed more repetitions to learn to solve the ToH problem and performed worse on the transfer tasks compared to participants with an induced positive mood.

In contrast to the findings showing that positive affect can facilitate problem-solving performance (e.g., Isen, 2001), some studies found that positive affect does not facilitate problems solving. In a study by Kaufmann and Vosburg (1997) high school students rated their affect at the beginning of the experiment and then engaged in solving insight problems which were unstructured and high in novelty and analytical tasks from an intelligence test. It was found that positive affect reduced problem-solving performance on the insight problems but not on the analytical tasks. These results were replicated in a second experiment with college students whose affect was induced using positive, negative, and neutral videotapes. The authors suggest that because students in their study did not receive any feedback and had to judge their solution for themselves, students with positive affect probably stopped searching for task-relevant information earlier than students with a negative mood. In line with this hypothesis, Spering et al. (2005) found that negative affect led to more detailed information search during complex problem-solving. In the study by Spering et al. (2005) with 74 undergraduate and graduate students, positive and negative affect were induced and the effect on complex problem-solving (CPS) was investigated. In CPS the situation is complex, variables are connected, there is a dynamic development of the situation, the situation is non-transparent, and people can pursue multiple goals (Funke, 2001). Positive and negative affect were induced by positive and negative performance feedback (Spering et al., 2005). Although, positive or negative affect increased as was intended, the results showed that positive and negative affect did not influence performance (Spering et al., 2005). However, negative affect did lead to more detailed information search and a more systematic approach (Spering et al., 2005).

To sum up, positive affect could facilitate problem-solving and decision-making. Yet, this seems to be dependent on the type of problems used in the different studies. The problem-solving tasks in the review by Isen (2001) were more structured or transparent than the ones used in the studies by Kaufmann and Vosburg (1997) and by Spering et al. (2005). For more structured problems, positive affect could facilitate problem-solving. If applied on learning to solve well-structured, stepwise hereditary problems in secondary education, one would expect positive affect to facilitate self-regulation of the learning process and problem-solving performance. The role of motivation, as described in the MASRL model by Efklides (2011) could interact with this process.

#### The Role of Motivation

Self-determination theory (SDT; Deci and Ryan, 2000; Ryan and Deci, 2000a,b) predicts that students use more effort and process the materials more deeply when they find the learning materials interesting. There are several types of motivation which can be placed on a continuum of the degree of experienced autonomy. Students with a high degree of autonomous motivation experience volition and psychological freedom. They study because the subject is interesting to them or it brings them satisfaction (i.e., intrinsic motivation). Also, doing the task could be valuable for attaining personal goals or development (i.e., identified motivation). However, students who score high on controlled motivation experience a low degree of autonomy and experience pressure. This pressure can come from within the student (i.e., introjected motivation). For example, students feel pressure to avoid feelings of shame, or pressure can come from an external source, such as demands from a teacher or a parent (i.e., external motivation).

Autonomous motivation types are associated with better learning outcomes, persistence, and psychological well-being relative to controlled motivation types. Autonomous motivation types were found to be related to better text comprehension (e.g., Vansteenkiste et al., 2004) and self-reported academic achievement (e.g., Vansteenkiste et al., 2009). Furthermore, motivation based on interest has been associated with better problem-solving performance (for a review see Mayer, 1998) and better SRL abilities such as effort regulation (i.e., controlling effort and attention) and metacognitive strategy use (i.e., checking and correcting one’s own learning behavior; Vansteenkiste et al., 2009). Moreover, it was found that students who indicated higher levels of interest for a course (i.e., an autonomous reason for studying), were more likely to use strategies to monitor and regulate their learning (Pintrich, 1999).

In summary, next to enhancing learning and problem-solving performance, autonomous motivation could also facilitate the use of SRL skills during learning. Furthermore, in multiple studies by Pekrun et al. (2002) intrinsic motivation was found to be related to positive affect such as enjoyment, hope, and pride. Also, negative affect such as boredom and hopelessness were found to be negatively related to intrinsic motivation and effort.

### Present Study and Hypotheses

The relation between affect, self-assessment accuracy, making complex decisions about the learning process (i.e., regulation choice complexity), perceived mental effect and motivation was investigated in a learner-controlled, online environment, in which students could monitor and regulate their own learning. In this environment students first received video-modeling examples teaching them how to solve stepwise, hereditary problem-solving tasks, how to make a self-assessment (i.e., monitoring), and how to select the next task (i.e., regulation choice). In each video-modeling example, after solving the problem, the model rated the perceived amount of invested mental effort (Paas, 1992), made a self-assessment of his/her performance over the five steps, made a regulation choice, and explained these actions (cf., Kostons et al., 2012; Raaijmakers et al., unpublished). After the video-modeling examples, students were asked to select and practice four problems from an overview with 75 problem-solving tasks. Affect was measured at the start of the study. Mental effort, self-assessment accuracy, and regulation choice complexity were measured during the posttest. Motivation was measured at the end or study.

**FIGURE 1:**
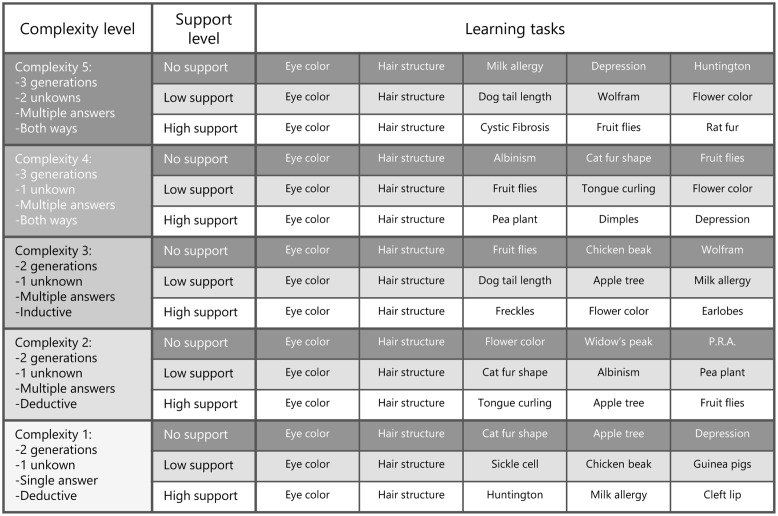
Task database containing the 75 problem-solving tasks showing the different levels of complexity, different levels of support, and the different surface features of the learning tasks (Raaijmakers et al., unpublished).

Although the problems in the learning phase were well-structured, the online learning environment in which students had to learn to solve them could be considered a complex problem-solving environment that required cognitive activities such as monitoring and planning with problem-solving tasks of different complexity levels (Osman, 2010). That is, during the learning phase students had to choose the problem-solving task they wanted to work on next from a task database with 75 tasks arranged by five complexity levels (see **Figure 1**). Task complexity of the well-structured problems was defined in terms of element interactivity: the higher the number of interacting information elements that a learner has to relate and keep active in WM when performing a task, the higher the complexity of that task and the higher the cognitive load it imposes (Sweller et al., 1998; Sweller, 2010). The easier problems consisted of less interacting information elements (e.g., two generations, one unknown, and deductive reasoning) compared to the more difficult problems (e.g., three generations, two unknowns, and both deductive and inductive reasoning). In addition, monitoring the learning process and choosing the next task at a certain complexity level based on monitoring processes also adds to the complexity of the learning process and imposes cognitive load upon the learner (e.g., Griffin et al., 2008; Van Gog et al., 2011). Taken together, monitoring learning and choosing tasks with different levels of interacting elements, created a complex problem-solving environment in which the current study took place.

We expected positive and negative affect, self-assessment accuracy, regulation choice complexity, perceived mental effort, and autonomous and controlled motivation to be predictors of problem-solving performance. More specifically, we expected positive affect measured at the beginning of the study to be a positive predictor of problem-solving performance (cf., Isen, 2001, Hypothesis 1a), whereas negative affect measured at the beginning of the study was expected to be a negative predictor of problem-solving performance (Hypothesis 1b).

According to theories of SRL (e.g., Winne and Hadwin, 1998; Zimmerman, 2008), we expected self-assessment accuracy during the posttest to be positively associated with problem-solving performance at the posttest (Hypothesis 2a). We further hypothesized that regulation choice complexity during the posttest would be positively associated with problem-solving performance at the posttest (Hypothesis 2b). Based on theories of SRL one would expect students to make regulation choices based on monitoring processes. Therefore, the more complex students’ regulation choices were, the better they think they performed (assuming that monitoring and regulation processes would approach actual performance and are more or less accurate).

Competition for WM resources between learning to solve a problem and self-regulation processes can have negative effects on either or both of these processes (Kanfer and Ackerman, 1989; Sweller et al., 1998). Based on the efficiency account of Paas and Van Merriënboer (1993; see also Van Gog and Paas, 2008) we assumed that the combination of perceived mental effort during the posttest and posttest performance would be indicative for the quality of learning (i.e., problem-solving) during the learning phase. Therefore, we hypothesized that students who managed to gain more knowledge during the learning phase, would experience lower mental effort during the posttest and obtain higher posttest performance than students who experience higher mental effort during the posttest. Therefore, perceived mental effort during the posttest was expected to be a negative predictor of problem-solving performance (Hypothesis 3a) and show a negative relation with SRL skills such as monitoring (Hypothesis 3b) and regulation choices (Hypothesis 3c) as measured during the posttest.

According to SDT, autonomous motivation is associated with better learning outcomes and SRL when compared to controlled motivation (Deci and Ryan, 2000). In line with the findings by Vansteenkiste et al. (2009), we expected autonomous motivation to be positively related to problem-solving performance (Hypothesis 4a), whereas controlled motivation was expected to be negatively related to problem-solving performance (Hypothesis 4b).

## Materials and Methods

### Participants

Participants were 136 secondary school students (*M*_age_ = 13.73, *SD* = 0.58, 74 girls) from the second year in the higher education track. All students gave their consent to participate in this study. Students’ parents received a letter in which information about the study was provided and parents were asked for their consent.

### Materials

Students participated in the computer rooms at their schools. They entered an online learning environment^1^ of which the content was created by the researchers for the purpose of this study. All measures were assessed online.

#### Affect Questionnaire

At the beginning of the study, all students filled out the 20-item Positive Affect and Negative Affect Scale (i.e., PANAS) on a 5-point scale (Watson et al., 1988). For both the positive affect scale (10-items) and the negative affect scale (10-items) an average score was calculated per participant. The reliability for the positive affect scale measured with Cronbach’s alpha was α = 0.76 and α = 0.76 for the negative affect scale.

#### Pretest and Posttest

The pretest and posttest consisted of three well-structured problem-solving tasks about hereditary problems based on the laws of Mendel which differed in complexity in terms of element interactivity (cf. Kostons et al., 2012). All problem-solving tasks consisted of five steps: (1) determining genotypes from phenotypes, (2) constructing a family tree, (3) determining whether the reasoning should be deductive or inductive, (4) filling out the crosstabs, (5) distracting the answer from the crosstabs (see Appendix A for an example). Problem-solving tasks 1 and 2 could both be solved by deducting the genotype of the child based on information about the parents. Task 2 was more difficult because the genotype of the parents was heterozygote vs. homozygote in task 1, which means that more interacting information elements needed to be taken into account during the problem-solving process. Problem-solving task 3 was the most complex problem-solving task because the genotype of one of the parents had to be induced based on information about the other parent and the child (i.e., inductive). This added more interactive information elements, and therefore complexity to the problem-solving process. The pretest and posttest were isomorphic to each other (i.e., different surface features were used). On both tests, students could score 1-point per correctly solved step adding up to 5-points per problem-solving tasks and 15-points in total.

#### Video-Modeling Examples

Two video-modeling examples showed how to solve a hereditary problem step by step. The hereditary problems explained in the videos had a similar solution procedure because in both videos the goal was to find the genotype of the child based on information about the parents (i.e., deductive). The surface features were different between the problems explained in the videos (i.e., nose bridge and tongue folding). In the videos, a model was thinking aloud about how to solve the problem and wrote down the solution step by step. One video had a female model and the other video had a male model explaining how to solve a problem (see Appendix B for an example). In each video after solving the problem, the model rated their mental effort on a 9-point scale (Paas, 1992), made a self-assessment of their performance over the five steps, made a regulation choice, and explained these actions (cf. Raaijmakers et al., unpublished). The regulation choice was based on a heuristic which uses performance and effort to choose the next task. The heuristic states that when one has a high performance combined with low mental effort one needs to choose a more difficult task, whereas with low performance and high effort one should choose an easier task (see Paas and Van Merriënboer, 1993; Van Gog and Paas, 2008).

#### Mental Effort Rating

After each posttest question, mental effort invested in solving the posttest problems was measured by asking: ‘*How much effort did you invest in solving this problem?*’ Students could respond on a 9-point scale, ranging from 1 (*very, very low mental effort*) to 9 (*very, very high mental effort*, Paas, 1992; Paas et al., 2003b; Van Gog et al., 2012). The mean mental effort rating for the pretest and the posttest was calculated. Unfortunately, six students did not fill out all the mental effort ratings and were left out of the analysis of the mental effort data (*n* = 130).

#### Self-assessment

Students made a self-assessment of their performance as a measure of self-monitoring after each posttest problem-solving task (cf. Baars et al., 2014a). Students rated which steps of the problem they thought they had solved correctly (0 indicating every step was wrong and 5 indicating every step was correct). Self-assessment accuracy was measured as absolute deviation (Schraw, 2009). Thus, absolute accuracy was calculated as the square root of the squared difference between actual performance and rated self-assessment per problem-solving task. The lower absolute deviation is, the smaller the distance between the self-assessment and the actual performance is and therefore, the more accurate self-monitoring (i.e., self-assessment) was. Unfortunately, six students did not fill out all the self-assessments and were left out of the analysis of the self-assessment data (*n* = 130).

#### Regulation Choice Complexity

During the posttest, the complexity of the regulation choices of students was measured. Students could choose problem-solving tasks to study next from a database with 75 problem-solving tasks at five complexity levels (see **Figure 1**, cf. Kostons et al., 2012; Raaijmakers et al., unpublished). They choose a task after solving each of the three posttest problems. The complexity of the regulation choice was measured with 1 being the easiest task to choose and 5 being the most difficult task to choose. The simplest problems consisted of 2 generations, 1 unknown, single answer, and deductive solution procedures. The most complex problems consisted of 3 generations, 2 unknowns, multiple answers, and deductive and inductive solutions procedures (for an overview see **Figure 1**). The level of support was not included in the level of complexity. Note, during the posttest students did not actually study the tasks they choose and they were made aware of that. The mean regulation choice complexity score for the posttest was calculated. There were 33 students who did not make a regulation choice and therefore they were left out of the analysis of regulation choice data (*n* = 103).

#### Motivation Questionnaire

At the end of the study, all students filled out a 16-item task-specific version of the academic self-regulation scale (Vansteenkiste et al., 2004). In four subscales, they had to indicate why they worked on solving the hereditary problem-solving tasks: (1) external (e.g., “… *because I am supposed to do so*”), (2) introjected (e.g., “… *because I would feel guilty if I did not do it*”), (3) identified (e.g., “… *because I could learn something from it*”), and (4) intrinsic motivation (e.g., “… *because I found it interesting*”). Items were measured on a 5-point Likert-type scale ranging from 1 (*not at all true*) to 5 (*totally true*). The four subscales were combined into an autonomous motivation composite (intrinsic and identified motivation) and a controlled motivation composite (introjected and external motivation; cf. Vansteenkiste et al., 2004). There were 10 students who did not complete the motivation questionnaire and therefore they were left out of the analysis of the motivation data. For the autonomous motivation composite (*n* = 126) Cronbach’s alpha was α = 0.89. For the controlled motivation composite (*n* = 126) Cronbach’s alpha was α = 0.65.

### Procedure

**FIGURE 2:**
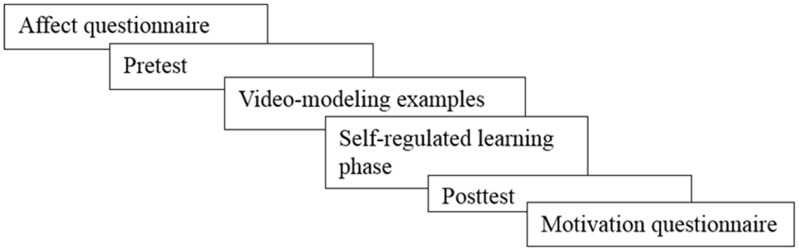
Procedure of the study.

In 50-min sessions in the computer room at their schools, students participated in the current study using an online learning environment^2^. In **Figure 2**, the procedure of the study is depicted. First, all students filled out the affect questionnaire. Then they took the pretest which was followed by two video-modeling examples. Then students entered the SRL phase in which they practiced with four problem-solving tasks of their choice from a database with 75 problem-solving tasks at five complexity levels (see the database in **Figure 1**). Students also practiced with rating their perceived mental effort, self-assessment, and regulation choices. Then after practicing four problem-solving tasks, students took a posttest with three problem-solving tasks of different complexity. Students’ perceived mental effort, self-assessments, regulation choices, and problem-solving performance were measured. Finally, all students filled out the motivation questionnaire.

## Results

In **Table 1**, the descriptive statistics of the pretest, posttest, perceived mental effort, self-assessments during the posttest (raw score, bias, and absolute accuracy), positive and negative affect scale, and autonomous and controlled motivation can be found. In **Table 2**, the correlations between these variables are shown. Pretest performance was significantly positively related to posttest performance. Positive affect was significantly positively associated with negative affect, indicating that students who scored higher on positive feelings also scored higher on negative feelings. Positive affect was significantly positively related to autonomous motivation. In line with Hypothesis 1b, negative affect was significantly negatively related to performance on the pretest and posttest, which indicated that students who reported more negative feelings scored lower on the tests.

**Table 1:** Means and Standard Deviations for performance, affect, motivation, and self-regulation variables.

Variable (Range)	*n*	Mean (*SD*)
Pretest score (0–15)	136	2.05 (1.59)
Perceived mental effort pretest (1–9)	136	7.74 (1.88)
Posttest score (0–15)	136	4.82 (2.85)
Perceived mental effort posttest (1–9)	130	5.60 (2.33)
Self-assessment rating (0–5)	130	2.71 (1.46)
Self-assessment bias (-5 to +5)	130	1.11 (1.60)
Self-assessment absolute accuracy (0–5)	130	1.74 (1.14)
Regulation complexity (1–5)	103	2.30 (1.26)
Positive affect scale (1–5)	136	2.64 (0.64)
Negative affect scale (1–5)	136	1.42 (0.44)
Autonomous motivation scale (1–5)	126	2.25 (0.84)
Controlled motivation scale (1–5)	126	2.70 (0.62)


**Table 2:** Summary of intercorrelations between measures.

Measures	1	2	3	4	5	6	7	8	9	10	11
(1) Positive affect	-										
(2) Negative affect	0.20*	-									
(3) Pretest performance	-0.07	-0.26**	-								
(4) Autonomous motivation	0.32**	-0.05	0.13	-							
(5) Controlled motivation	0.12	0.02	0.02	0.23**	-						
(6) Mental effort (posttest)	-0.03	0.11	-0.01	-0.09	0.16	-					
(7) Self-assessment raw score	0.07	-0.01	0.07	0.12	-0.08	-0.53**	-				
(8) Self-assessment bias	0.03	0.16	-0.12	-0.07	-0.10	-0.32**	0.81**	-			
(9) Self-assessment absolute accuracy	-0.04	0.15	-0.11	-0.18*	-0.03	-0.12	0.63**	0.81**	-		
(10) Regulation choice complexity	-0.07	-0.00	-0.01	0.06	-0.12	-0.30**	0.27**	0.23*	0.16	-	
(11) Posttest performance	0.06	-0.22**	0.29**	0.30**	0.05	-0.27**	0.17	-0.44**	-0.40**	0.04	-


*^∗^Correlation is significant at the 0.05 level (2-tailed). ^∗∗^Correlation is significant at the 0.01 level (2-tailed). High values for self-assessment absolute accuracy indicate low accuracy.*

In line with Hypothesis 2a, both self-assessment bias and absolute accuracy of self-assessments during the posttest were significantly negatively related to posttest performance. That is, the larger the difference between self-assessment and actual performance was, the lower posttest performance was. It seemed that students who are less accurate in their self-assessment also score lower on the posttest.

In support of Hypotheses 3b and c, the ratings of perceived mental effort showed a significant negative relation with the self-assessment raw score, bias, and complexity of regulation choices. This means that students who experienced a higher mental effort showed lower self-assessment and bias values, and choose less complex tasks to restudy. Both self-assessment raw scores and bias were positively correlated to the complexity of regulation choices. That is, the higher self-assessment raw scores and bias were, the more complex regulation choices were. In line with theories of SRL, this shows the sensitivity of regulation choices in relation to self-assessments (control sensitivity; Koriat and Goldsmith, 1996). Also, in line with Hypothesis 3a, perceived mental effort was significantly negatively related to posttest performance.

Autonomous motivation was significantly positively related to controlled motivation. It seems that students who scored higher on autonomous motivation also scored higher on the controlled motivation. Autonomous motivation also showed a significant negative relation with self-assessment absolute accuracy during the posttest. That is, students who scored higher on autonomous motivation had lower absolute accuracy scores which means that the deviation between their self-assessment and actual posttest performance was smaller. In other words, students with higher autonomous motivation also had more accurate self-assessments during the posttest. In support of Hypothesis 4b, autonomous motivation also showed a significant positive relation with posttest performance. This indicates that students who scored higher on autonomous motivation also scored higher on the posttest.

### Regulation Choices and Problem Complexity

The complexity level at which students selected a task for restudy and how they performed on the different complexity levels in the posttest were explored. Regulation choice complexity was not normally distributed. The mode of all three selection moments was regulation choice complexity 1. Therefore, a Friedman’s ANOVA was conducted for regulation choice complexity at all three selection moments during the posttest. The regulation choice complexity differed significantly over the three moments, χ^2^(2) = 8.59, *p* = 0.014. Wilcoxon tests were used to follow up this finding. It appeared that regulation choice complexity differed significantly between moments 1 (*Mean rank* = 2.14) and 3 (*Mean rank* = 1.83), *T* = 0.30, *r* = 0.21 (small effect size). Yet, no significant differences between selection moments 1 and 2 (*Mean rank* = 2.03) or 2 and 3 were found.

Furthermore, as a check on the complexity of the problem-solving tasks in terms of element interactivity, a repeated measures ANOVA with complexity levels as a within-subjects variable was performed. It showed that problem-solving performance on the posttest differed significantly between the complexity levels of the problem-solving tasks, *F*(1,135) = 56.13, *p* < 0.001, $\eta_{p}^{2}$ = 0.30. Performance on the least complex problem-solving task 1 (*M* = 2.45, *SD* = 1.68) was significantly higher compared to task 2 (*M* = 1.23, *SD* = 1.40), *p* < 0.001, and compared to task 3 (*M* = 1.14, *SD* = 0.71), *p* < 0.001. There was no significant difference between performance on tasks 2 and 3.

### Affect, SRL Skills, and Motivation As Predictors for Problem-Solving Performance

We performed stepwise regression with pretest performance in Model 1 and positive affect, negative affect, self-assessment accuracy during the posttest, regulation choice complexity, perceived mental effort, autonomous, and controlled motivation in Model 2. We assessed multicollinearity in accordance with the guidelines by Field (2009) by checking the VIF and tolerance values. The VIF provides an indication of whether a predictor has a strong relationship with the other predictor(s) and the tolerance statistic is defined as 1/VIF. VIF values were well below 10 and tolerance was well above 0.2. Thus, collinearity was not a problem for our model (Field, 2009).

As shown in **Table 3**, Model 1 with pretest performance as a predictor of posttest problem-solving performance was significant, *F*(1,100) = 8.80, *p* = 0.004, *R*^2^ = 0.08. Pretest performance was a significant positive predictor of posttest problem-solving performance.

**Table 3:** Stepwise regression with predictors of problem-solving performance.

	*b*	*SE*	β	*p*
**Step 1**				
Constant	3.85	0.44		<0.001
Pretest performance	0.50	0.17	0.28	0.004
**Step 2**				
Constant	6.05	1.89		0.002
Pretest performance	0.34	0.16	0.19	0.035
Positive affect	0.49	0.42	0.11	0.252
Negative affect	-1.54	0.76	-0.19	0.046
Self-assessment accuracy	-0.83	0.24	-0.31	0.001
Regulation choice complexity	0.06	0.21	0.03	0.768
Mental effort posttest	-0.29	0.12	-0.24	0.013
Autonomous motivation	0.41	0.34	0.12	0.232
Controlled motivation	0.34	0.46	0.07	0.464


**R*^2^ = 0.08 for Step 1; Δ*R*^2^ = 0.30 for Step 2 (*p* = 0.001).*

In Model 2, positive affect, negative affect, posttest self-assessment accuracy, posttest regulation choice complexity, posttest perceived mental effort, autonomous and controlled motivation were added as predictors, *F*(8,93) = 4.89, *p* < 0.001, *R*^2^ = 0.30. Model 2 explained more variance compared to Model 1, Δ*R*^2^ = 0.22, *p* = 0.001. Pretest performance was again a significant positive predictor or posttest problem-solving performance in Model 2. In line with Hypothesis 1b, negative affect was a significant negative predictor of posttest problem-solving performance. That is, the more negative affect students reported, the lower their posttest performance was. Also, in support of Hypothesis 2a, self-assessment accuracy was a significant negative predictor of posttest problem-solving performance. Self-assessment accuracy during the posttest was measured as absolute accuracy. The lower this measure is the more accurate self-assessments were. Thus, the negative relation with posttest performance means that the less accurate students’ self-assessments were, the lower posttest performance was. Furthermore, in line with Hypothesis 3, perceived mental effort was a significant negative predictor of posttest problem-solving performance. That is, the higher perceived mental effort during the posttest was, the lower posttest problem-solving performance was.

## Conclusion and Discussion

The current study investigated the relation between affect (i.e., positive affect and negative affect), SRL skills (i.e., monitoring and regulation), perceived mental effort, motivation (i.e., autonomous and controlled motivation), and performance when learning to solve problems in a complex learner-controlled, online learning environment with secondary education students. Students performed worse on the more complex problems during the posttest. Also, regulation choice complexity was lower after the most difficult problem-solving task when compared to the least complex problem-solving task at the posttest. Interestingly, the results showed that students’ negative affect, SRL skills, and perceived mental effort play a crucial role in learning to solve problems in a self-regulated way in a learner-controlled study environment.

In contrast to Hypothesis 1a, positive affect was not a significant predictor of problem-solving performance in the current study using well-structured problem-solving tasks with high element interactivity. This result does not fit previous findings showing that positive affect improves cognitive processing (e.g., Estrada et al., 1994; Isen, 2001; Brand et al., 2007; Politis and Houtz, 2015) and academic achievement (Mega et al., 2014). Possibly, this difference can be explained by the way affect was measured and whether it was induced or not. Many of the studies reviewed by Isen (2001), in the studies by Estrada et al. (1994), Brand et al. (2007), and Politis and Houtz (2015) induced positive affect was found to improve different aspects of problem-solving performance. In the current study, positive affect was measured using a questionnaire at the beginning of the study. Therefore, it could be that positive affect measured by a rating provided by students does not have the same effect as induced positive affect on problem-solving performance. The effect of positive affect without inducement might be more prominent on more general measures of achievement made over a period of time (e.g., Mega et al., 2014). Interestingly, in the study by Kaufmann and Vosburg (1997) high school students also rated their affect and it was found that positive affect reduced performance on insight problems but not on analytical tasks. Yet, in our study we did not find a positive or negative association of positive affect with problem-solving performance on well-structured stepwise problems in high school. Furthermore, in our study students learned to solve problems in a self-regulated way and had to make decisions about which tasks to practice which made the learning process as a whole quite complex for students. Spering et al. (2005) found that in CPS, performance was not affected by positive or negative affect which would be partially in line with our findings (i.e., no relation between positive affect and problem-solving performance). Yet, in line with earlier results (e.g., Pekrun et al., 2002; Mega et al., 2014) and our hypothesis, we found that negative affect influenced problem-solving performance. Specifically, in support of Hypothesis 1b, negative affect negatively predicted problem-solving performance. The difference between the results found by Kaufmann and Vosburg (1997) and the current study might be explained by the difference in the type of problem-solving tasks used in both studies. Although, element interactivity made the problems complex for students, the stepwise solving procedure also made the problem-solving tasks well-structured. Possibly, our problem-solving tasks were more transparent and therefore less complex than the insight problems used by Kaufmann and Vosburg (1997). Because of different dimension on which complexity can be defined (e.g., structure, element interactivity, and transparency), future research should investigate the relation of positive and negative affect with these different dimensions of complexity in problem-solving tasks.

In line with Hypothesis 2a and theories of SRL (e.g., Winne and Hadwin, 1998; Zimmerman, 2008), self-assessment accuracy was positively related to problem-solving performance. Students who were less accurate in their self-assessments, showed lower posttest problem-solving performance. Hence, monitoring seems an important prerequisite for successful learning to solve problems in a self-regulated way. However, there is a possibility that students who were high performers, were also better able to monitor their own learning. The results of the current study cannot establish the causality of this relation. Future research could use an experiment to investigate the effect of monitoring on problem-solving performance.

In contrast to Hypothesis 2b, the regulation choice complexity was not related to problem-solving performance. This might be explained by the way we operationalized regulation choices. That is, students had to choose what task they wanted to work on next. According to the discrepancy-reduction framework of regulation (e.g., Nelson et al., 1994) students would choose tasks in between their current state of learning and the goal state. Within this perspective on regulation of learning, choosing more difficult tasks would contribute to successful SRL. Yet, students might have chosen to select a task they were almost able to solve, which would be in line with the region of proximal learning to explain regulation of learning (e.g., Kornell and Metcalfe, 2006). Also, students might have chosen the task because they were curious about or just wanted to solve based on an agenda they might have had for themselves (i.e., agenda-based regulation, Ariel et al., 2009). For example, students could have been curious about the most complex problems or they wanted to finish as fast as possible and therefore choose the easiest problems. Also, regulation choices might have been inaccurate (i.e., deviate from actual performance). If students were not able to accurately monitor and/or regulate their own learning, regulation choice complexity would not be related to performance (cf. Baars et al., 2014a, 2016). This could also be caused by the fact that the regulation choices made during the posttest were not granted (i.e., students did not actually work on the problem they chose again). Future research could investigate the reasons students have to choose certain tasks to regulate their learning and if these choices are accurate in relation to their performance. In addition, future research could grant students their regulation choices and investigate how that would affect subsequent problem-solving performance.

Perceived mental effort during the posttest was significantly related to problem-solving performance which was in line with Hypothesis 3. That is, the more mental effort students experienced during the posttest, the lower their posttest performance was. This finding is in line with CLT (e.g., Sweller et al., 1998) and the efficiency account introduced by Paas and Van Merriënboer (1993; see also Van Gog and Paas, 2008). Yet, it would be interesting to follow up on this finding by including measures of perceived mental effort and performance during the learning phase in future research. That way the learning process and the relation to perceived mental effort could be investigated more elaborately. Furthermore, in the current study perceived mental effort was also related to the complexity of regulation choices during the posttest. That is, students who experienced higher mental effort during the posttest, chose less complex problems when making regulation choices during the posttest. Possibly, students used their perceived mental effort as an indicator to regulate their learning. This is in line with earlier research showing that students use their study effort to regulate their learning when regulation is data-driven (i.e., based on the ease of learning, Koriat et al., 2014). This seems sensible because mental effort was a significant predictor of problem-solving performance. This result provides support for studies showing that training students to use their perceived mental effort to regulate their learning when learning to solve problems can be effective (e.g., Kostons et al., 2012; Raaijmakers et al., unpublished). Future research could also include measures of perceived difficulty and self-efficacy to investigate the relation between perceived mental effort, task difficulty, self-efficacy, and performance during SRL.

Contrary to our expectations (Hypotheses 4a and 4b), motivation was not a significant predictor of problem-solving performance. Based on earlier studies (e.g., Vansteenkiste et al., 2009) autonomous motivation was expected to be a positive predictor of problem-solving performance. Yet, in the current study we did not investigate the different types of motivation (i.e., profiles: autonomous, identified, introjected, and external motivation, Vansteenkiste et al., 2009). Perhaps when taking into account the differences between motivational profiles, the effect of motivation on performance would be more pronounced. In addition, motivation was measured at the end of the experiment because students needed to be familiar with the materials used in the study. Yet, perhaps because of fatigue or boredom, students rated their motivation lower at the end of study compared to a measurement that would have been earlier on in the study. Future research could investigate this by placing the motivation questionnaire right after the pretest which would give students an idea of the materials without being mentally exhausted.

Limitations of the current study are the small number of secondary education students who could take part in the study. Future research could replicate the current study with more participants. This would also enable researchers to take into account different motivational profiles and their relation to positive and negative affect as predictors of problem-solving performance. Also, problem-solving performance was quite low and measured using a limited set of tasks during the posttest. It would be interesting to use more tasks for a longer period of time covering the SRL phase and posttest to investigate the effect of motivation and affect. We found positive and negative affect to be positively related which could have been caused by the intensity of affect (Diener et al., 1985). Future research could measure this dimension of affect to control for it. In addition, for both motivation and affect questionnaires were used as a measurement. The motivation questionnaire was task specific and therefore placed at the end of the study, which could have caused students to use their experience of success or failure during the posttest when filling out the motivation questionnaire. Future research could design experiments in which affect is induced and motivation is measured earlier during the study or through the learning behaviors of students.

In conclusion, the current study showed that negative affect, monitoring accuracy, and perceived mental effort are predictors of problem-solving performance of secondary education students learning to solve problems in a learner-controlled, online environment. The fact that these predictors were all negatively related to performance is an important indication that students need more support when learning to solve problems in a self-regulated way. Interventions to support SRL processes (e.g., training, cf. Kostons et al., 2012) and reduce mental effort involved in learning to solve problems (e.g., worked-examples, Sweller, 1988), could potentially prevent negative effects of inaccurate monitoring and too high cognitive load during learning. Future research could investigate the role of support during learning to solve problems in a self-regulated way.

## Ethics Statement

In accordance with the guidelines of the ethical committee at the Department of Psychology, Education and Child studies, Erasmus University Rotterdam, the study was exempt from ethical approval procedures because the materials and procedures were not invasive.

## Author Contributions

MB, LW, and FP worked together on the theoretical framework and the design of the study. Both MB and LW prepared the materials, and collected the data. MB, LW, and FP worked together on the analysis of the data. Writing the manuscript was done by MB, LW, and FP in collaboration. Finally, MB, LW, and FP approved the manuscript and are accountable for it.

## Conflict of Interest Statement

The authors declare that the research was conducted in the absence of any commercial or financial relationships that could be construed as a potential conflict of interest.

## Footnotes

1 www.qualtrics.com

2 www.qualtrics.com
