# Supplementary material for: The Association between Motivation, Affect, and Self-regulated Learning When Solving Problems
Source: Front Psychol. 2017 Aug 8;8:1346. doi: 10.3389/fpsyg.2017.01346 (PMC5550677; doi:10.3389/fpsyg.2017.01346)

**Appendix A**

*Example of problem-solving task used in pretest and posttest (first level of complexity)*

**Fur color**

A guinea pig’s fur color is determined by a gene, which expresses itself as black in its dominant form (F) and white in its recessive form (f). Two guinea pigs, who are both black and homozygote for that trait, produce offspring. What are the possible genotypes for this offspring?

Step 1. Translate information from text into genotypes.

Mother: ……………………….

Father: ……………………….

Step 2. Fill in a family tree.


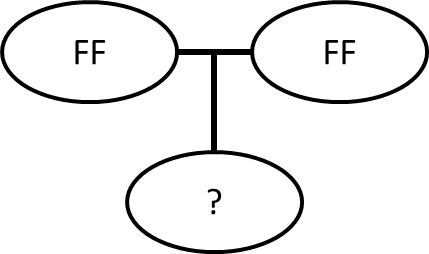


Step 3. Determine number of Punnett squares by deciding if problem is to be solved deductively or inductively.

……………………….

Step 4. Fill in the Punnett square.

|  |  |  |
| --- | --- | --- |
|  |  |  |
|  |  |  |

Step 5. Find the answer in the Punnett square. Which of the following possibilities is the right answer?

- Ff
- FF
- Ff
- FF and Ff
- FF and ff
- Ff and Ff
- FF, Ff, and ff

**Appendix B**

A still from one of the video modeling examples showing how the model is able to write out the problem-solving steps underneath the problem statement.


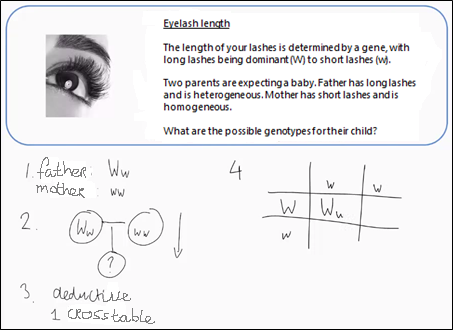

Supplement: Supplementary file 1 [file Data_Sheet_1.docx]
